# Supplementary material for: Comparative multiomics analysis of cell physiological state after culture in a basket bioreactor
Source: Sci Rep. 2022 Nov 23;12:20161. doi: 10.1038/s41598-022-24687-4 (PMC9686226; doi:10.1038/s41598-022-24687-4)

6037.1:192246952:192247140:+@NW_023666037.1:192246952:192247220:+@NW_023666037.1:192248093:192

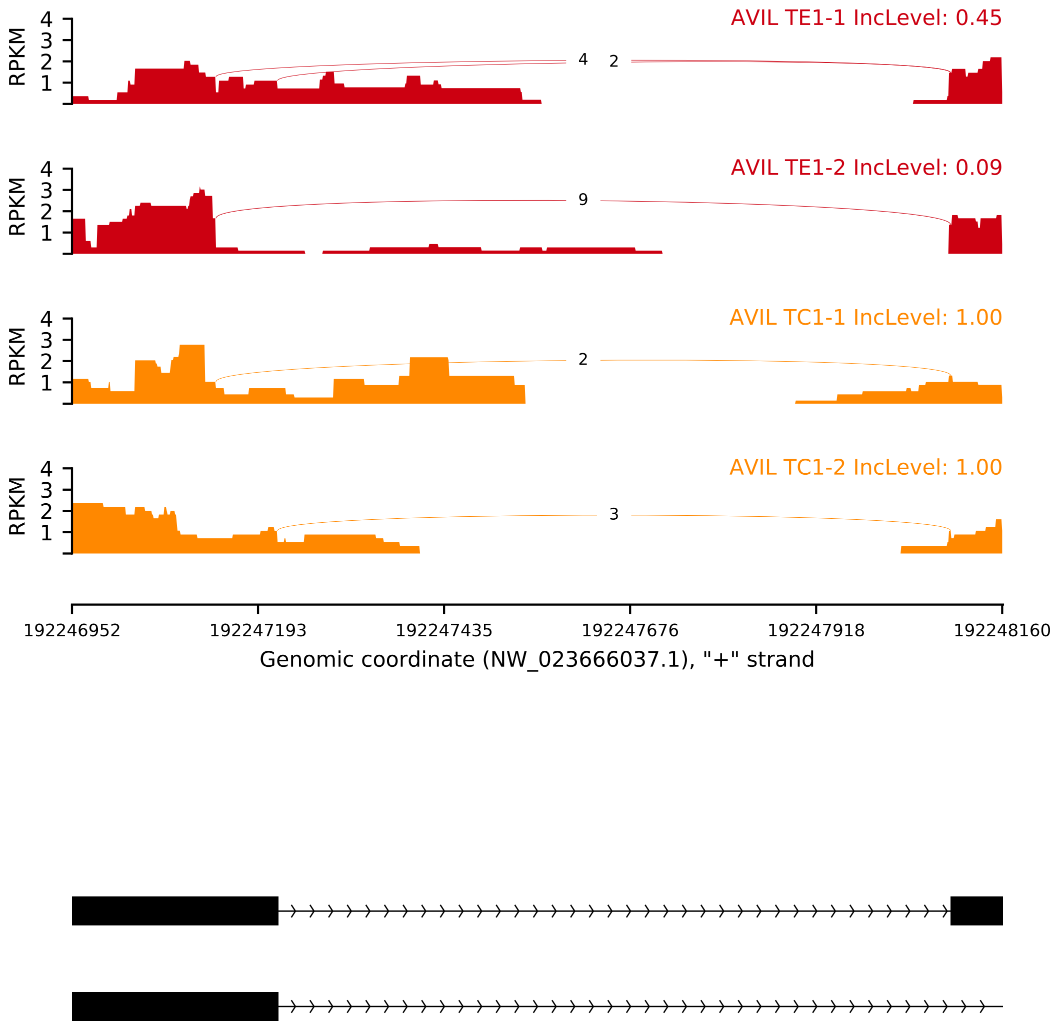

Supplement: Supplementary file 1 — Supplementary Information 1. [file 41598_2022_24687_MOESM1_ESM.zip › raw data/Transcriptomic raw data/7.AS/2.dasplot/TE1vsTC1/A5SS/13_AVIL_NW_023666037.1_192246952_192247140_+@NW_023666037.1_192246952_192247220_+@NW_023666037.1_192248093_192248161_+.pdf]
